# Effect of Natural Fiber and Biomass on Acoustic Performance of 3D Hybrid Fabric-Reinforced Composite Panels

**DOI:** 10.3390/ma17235695

**Published:** 2024-11-21

**Authors:** Shabnam Nazari, Tatiana Alexiou Ivanova, Rajesh Kumar Mishra, Miroslav Müller, Mehdi Akhbari, Zohreh Esfahani Hashjin

**Affiliations:** 1 Department of Sustainable Technologies, Faculty of Tropical AgriSciences, Czech University of Life Sciences Prague, Kamycka 129, Suchdol, 165 00 Prague, Czech Republic; nazari@ftz.czu.cz; 2 Department of Material Science and Manufacturing Technology, Faculty of Engineering, Czech University of Life Sciences Prague, Kamycka 129, Suchdol, 165 00 Prague, Czech Republic; muller@tf.czu.cz; 3 Sialk Industrial Innovators, o. 1, Daman Afshar Alley, Unit 6, Africa Blvd., Tehran P.O. Box 13, Iran; meha49534953@gmail.com; 4 Razi Metallurgical Research Center, No. 8, Fernan St., HajGhasem Asghari Blvd., Shahre Ghods Entrance (Sorkhe Hesar), Tehran P.O. Box 39, Iran; zohrehesfahani88@gmail.com

**Keywords:** eco-friendly construction materials, sustainability, biomass utilization, 3D spacer panels, acoustic insulation

## Abstract

This research investigated the sound insulation performance of 3D woven hybrid fabric-reinforced composites using natural fibers, such as jute, along with E-glass and biomass derived from agro-waste, e.g., coffee husk and waste palm fiber. The composites made from pure E-glass, pure jute, and hybrid glass–jute configurations were tested for sound absorbance at frequencies of 1000 Hz and 10,000 Hz. A sound insulation chamber was used for measuring the sound reduction levels. Results show that the sound insulation performance of the panels was remarkably enhanced with composites containing natural fiber reinforcements. The jute-based composites provided the maximum insulation of sound, with waste palm fiber fillers in particular. At a frequency of 10,000 Hz, a noise reduction reaching 44.9 dB was observed. The highest sound absorption was observed in the 3D woven jute composites with the additive of waste palm fiber, which outperformed the other samples. When comparing the effect of coffee husk and palm fiber as biomass fillers, both exhibited notable improvements in sound insulation, but the palm fiber generally performed better across different samples. Although panels containing palm fiber additives appeared to reduce sound more than those containing coffee husk, statistical analysis revealed no significant difference between the two, indicating that both are efficient and eco-friendly fillers for soundproofing applications. One-way analysis of variance (ANOVA) confirmed the significance of the effect of reinforcing structures and biofillers on acoustic performance. This study demonstrated the possibility of using sustainable green materials for soundproofing applications within various industries.

## 1. Introduction

Most of the industrial machinery designed for high-speed transit or for improving living conditions produces a lot of noise, which is an undesirable level of sound [1]. Noise is one of the four main pollution categories in the globe, apart from air, water, and land pollution. Regular noise exposure can lead to several health issues, including heart disease, sleep disorders, and hearing loss [2]. Recently, there has been a lot of focus on noise management as a way to improve living conditions [3]. The goal of transportation planners, acoustical engineers, and architects is always to develop innovative methods to cut down on or eliminate noise. Their task is to preserve or improve the visual environment while still achieving the intended low sound levels [4]. In recent years, nonwovens and felts have been the most investigated textile materials for sound absorption [5].

Due to their interlaced structures, woven fabrics exhibit relatively high strength and low wear performance, making them highly durable. Woven textiles remain intact longer while functioning in the application areas without much degradation. Because of these traits, they are especially fit for use in high-strength stable service applications, including automotive interiors, architectural panels, and industrial equipment [6]. As the yarns are interlaced with each other, the mechanical behavior of woven fabric structures is typically better than other types of structures, e.g., knitted or nonwovens [7,8]. However, woven textiles underperform when it comes to sound absorption capacities [9].

Because of their smaller thickness, woven textiles have lower sound absorption capacity than nonwoven and fiber-based felts. However, because of their strong structural design ability and dimensional stability, woven fabrics are typically employed in automotive decorations and domestic textiles [10,11].

Traditional sound-absorbing materials such as fiberglass and mineral wool are widely used due to their high sound absorption across a broad frequency range. Recent developments in natural fiber-based composites, including those utilizing jute, coffee husk, and palm fibers, provide eco-friendly alternatives with competitive noise reduction coefficients [12]. Studies on 3D woven spacer fabrics also demonstrate their effectiveness in acoustic insulation due to their porous and multi-layered structure, which enhances sound absorption, particularly in mid-to-high frequency ranges [6,13]. These advancements underscore the potential of sustainable composites as viable substitutes for traditional materials, combining environmental benefits with soundproofing efficiency.

For the category of general composites, 3D textiles have a special structural advantage due to the multi-layered structure with vertical linkages. This includes two interwoven surfaces bridged by vertical piles, with considerable numbers of interstitial spaces in the fabric that can serve to trap and dissipate sound waves effectively. This provides better acoustic insulation than that possible with conventional 2D composites. Furthermore, the porosity and flexibility within 3D textiles enhance sound-scattering and absorption capability to reduce internal material vibration transmission. Due to their multi-layered and interconnected structural nature, 3D textiles have specific benefits for thermal insulation and also offer potential benefits in the acoustic domain. Such interstitial spaces make it more efficient for sound waves to be trapped and scattered, reducing the transmission of vibrations [14,15,16]. Natural fibers such as jute, when used within 3D textiles, further enhance these sound-insulating properties by offering better damping of vibrations than synthetic materials.

These features make the 3D woven textiles very suitable for applications that require efficient soundproofing, like interior building, automotive parts, and industrial machinery [17]. It is reported that three-dimensional (3D) textile structural composites offer an exceptional integrated structure and strong resistance to delamination, making them competitive materials for use in the communication, automotive, and aerospace industries [8]. However, as composites have advanced, the focus of design has steadily switched from concentrating primarily on mechanical qualities to multifunctional integration. The “focus on design” refers to using innovative techniques in composite materials to enhance both mechanical properties and sound insulation capabilities. These design improvements are particularly effective in natural fiber-reinforced composites, like jute and glass fiber, allowing for multifunctional applications in acoustic insulation [9]. Natural fiber-reinforced thermoplastic composites have advanced rapidly in recent decades with the goal of creating a new class of composites that are renewable, biodegradable, and capable of storing carbon [18,19]. Polylactic acid (PLA)-based composites are a possible alternative for achieving these objectives. However, there are certain issues related to PLA in composites. The intrinsic brittleness of PLA polymer is enhanced when natural fibers are cut into small pieces for use as reinforcing materials [20]. The random orientation of the fibers in the composite and the fiber mechanical damage during common shear mixing procedures like injection molding prevent the effective exploitation of the fiber’s mechanical potential [21]. Therefore, structurally integrated 3D textile composites with PLA can be the ideal solution to overcome such issues.

However, researchers suggest that textile fabrics with specific fiber orientations might be employed as reinforcement to improve the mechanical performance of composites [22]. One of the most promising types of textile composites for advanced applications is the three-dimensional orthogonal woven composite, which offers enhanced structural integrity due to its unique interwoven design. Three distinct fiber orientations are present in composite materials: longitudinal, transverse, and through-thickness. In the case of woven constructions, this 3D alignment improves the acoustic insulation and interlaminar strength. Such orientation will ensure that the fibers are along the X-Y-Z axes, which is crucial for achieving the enhancement of mechanical stability and soundproof properties of the composite. It maintains comparatively excellent in-plane mechanical characteristics due to its crimp-free yarn form, which also provides exceptional damage tolerance and delamination resistance [23,24,25]. This improves the stability and performance compared to two-dimensional woven laminated composites. According to the literature, thermosetting resin composites reinforced by high-performance fibers such as carbon and glass fibers have accounted for the majority of published research on 3D composites [2,26].

Natural fibers, also referred to as cellulose fibers, are environmentally benign and made of nearly renewable resources. The use of cellulose fibers as reinforcement in composite materials has sparked interest again in recent years. This is because cellulose fibers—found in jute, coir, sisal, and other materials—have a number of advantages over synthetic fibers, including the ability to withstand wear and tear, a low density, an acceptable specific strength, superior thermal and insulating properties, require less energy, are more affordable, and biodegrade [27]. Jute is a well-researched natural fiber that has a higher cellulose content than other natural fibers [28]. Natural plant fibers are becoming more popular as an inexpensive engineering material for fiber-reinforced plastics (FRP) because of their characteristics, such as high specific strength, low density, and renewability. Furthermore, industrial sectors are under pressure from consumers and new environmental laws to find alternative materials that can take the place of traditional, non-renewable reinforcing materials like glass fiber. Commodity composites made of natural fibers are mostly used because they are inexpensive and have usable structural qualities [29,30,31]. Because of their unique structure, natural fibers and their composites have drawn more attention recently as potential insulators and sound absorbers [32,33]. Without a doubt, hybrid structural design offers a practical way to reduce material costs while enhancing material performance overall [34,35].

Despite having relatively poor thermal insulation qualities, traditional organic fibers like jute, bamboo, and wood fiberboard can be used as sound absorption materials and perform well in this regard. However, these materials are also characterized by their lower flame resistance, poor thermal stability, and sensitivity to ambient humidity [36]. When compared to organic materials, inorganic fiber—which primarily consists of glass wool and mineral wool—has a higher sound absorption coefficient and performs better as a thermal insulator. But because these fibers are brittle and delicate, they can break down into powder that irritates and impedes breathing. With their integrated structure and overall performance, three-dimensional looms weave fabrics with a great deal of promise for use in aerospace, marine, military, and other areas [37]. A well-thought-out structural design can increase the 3D fabric-reinforced composite’s weight and volume, which can broaden its use in the areas of heat insulation and sound absorption [38]. On the other hand, not much is known about the 3D fabric-reinforced composites’ ability to insulate heat and absorb sound [39].

Acoustic methods are becoming popular in the evaluation and characterization of both 2D and 3D embroidered fabrics because of their non-invasiveness and high precision. Such methods involve sending sound waves through or against fabric materials and measuring the resulting patterns. Changes in these parameters provide information about the texture, porosity, and density of woven or non-woven fabrics [37,38]. For example, tension, elasticity, or discretion to external forces are some of the parameters that can be determined using acoustic ways in 2D fabric structures, and these contribute immensely towards knowing the strength and flexibility of the fabric material. Extending these methods provides more advantages, especially in assessing 3D fabrics by considering the structural configuration and the arrangement of fibers in the space and, hence, evaluating bulk properties such as thickness and compressibility of layers of the material. Since sound travels at a different speed in the presence of gouges or scratches within the fabric layers, such fabrics can be used in sound wave-based quality control for advanced composite fabrics in the aerospace, automotive, and protective clothing industries. Also, using modern fabrication techniques such as noise mapping makes it possible to build better models for simulating the response of fabrics to unknown conditions [37,38,39].

Although there are numerous research studies about natural fiber-reinforced polymer composites, there are only a few focused on hybrid woven fabric composites for acoustic performance. The composites in this study are made from a combination of jute and E-glass fibers with waste fibers as surface fillers. The 3D reinforcing structure in the composites is intertwined in many layers, and this fact brings enhanced mechanical properties, such as strength and durability, as well as better sound absorbance.

Based on the evidence in the literature, the objective of this investigation is to assess the possibilities of incorporating natural fibers like jute and waste agricultural fibers such as coffee husk and palm fiber fibers to enhance the sound insulation characteristics of 3D hybrid woven fabric-reinforced polymer composites. With this inclination toward the acoustic potential of such materials, this study aims to find out how the use of biodegradable fibers and fillers will affect the sound intensity in composite materials. This study is also relevant in addressing the need for new promising materials that are functional and environmentally friendly, especially in the areas where thermal and sound insulation materials are used. Our results show that natural and waste biomass fibers can serve as composite fillers not only as lightweight and cheaper alternatives to structural composites but also for their great sound-absorbing capabilities. By systematically evaluating the sound insulation parameters of these fiber-reinforced composites, this study contributes to the field of sustainable materials research, offering insights into how eco-friendly fibers can reduce the environmental impact while maintaining or even improving performance in sound insulation applications. This work brings out the aspect of environmental sustainability along with functional efficiency in the quest for newer composite materials. The possibility of a longer lifetime and retarded degradation is given in comparison with conventional 2D composites.

## 2. Materials and Methods

### 2.1. Materials

E-glass yarns were utilized in structural fabrics. E-glass is the most adaptable, most economical, and most utilized fiber in different applications. E-glass has been chosen because of its outstanding resistance to environmental elements, excellent tensile strength, and relatively cheap price. Presently, E-glass is the most widely used reinforcement synthetic in composites, accounting for almost 90% of the market share in synthetic fiber composites. E-glass is more feasible than standard glass fibers, which are brittle and tend to break under flexion forces, due to its flexibility during weaving, reducing the possibility of breakage.

Jute is one of the least expensive natural fibers and ranks second only to cotton in global production. It belongs to the bast fiber group. It is completely biodegradable, recyclable, and therefore eco-friendly. Jute was chosen as the natural fiber fraction because it is adaptable and has high tensile strength, making it easy to be woven into fabric. During weaving, E-glass is much stronger and less likely to rupture than standard glass fibers. The high tensile strength of E-glass, coupled with the pliability of jute, increases weaving efficiency. The weaving efficiency was minimized in this research as the focus was the use of green materials.

Glass threads and jute were utilized to create 3D fabrics in the study, producing jute/glass fiber hybrid spacer fabric (3DKJGF). The glass fibers had a linear density of 600 Tex, while the jute threads had a linear density of 550 Tex (g/km). Next, preforms were prepared and saturated with ortho-phthalic unsaturated polyester resin (B-222) and hardener using a manual layup method. Fabric type, thickness, weft density, and yarn fineness (linear density) for E-glass, jute, and pile yarns were analyzed to produce 3D woven fabrics using a modified face-to-face carpet weaving machine. Vertical piles connect two bi-directional woven fabrics serving as face sheets. The pile yarns are attached to the front sheet in the warp direction in an ‘8’ shape and in the weft direction in a ‘c’ shape. In this research, samples of three types of fabrics—glass (3DGF), jute (3DJF), and hybrid (3DJGF)—were produced with fixed weave parameters by Novavaran Sanat Silk Co., Ltd., Kashan, Iran. These 3D fabrics eliminate delamination, allowing precise control of the composite material properties by manipulating the characteristics of warp, weft, pile yarns, and fabric structure. This company can produce 3D glass fabrics with unlimited length and a maximum width of 4 m. The structural properties and types of fabrics are detailed in Table 1.

This table highlights the structural properties and fiber types of three different 3D woven fabrics: 3DGF, 3DJF, and 3DJGF. All types of fabrics were prepared with 2.7 mm structural thickness and had a weft density of 3.2 yarns per centimeter, demonstrating uniformity in the construction of fabric types. From these measurements, it can be implied that the fabrics have been manufactured aiming to achieve “systematic” material development. The only departure from this uniformity would be through the changes in the fiber material. There would be changes in mechanical behavior, durability, and performance of the fabric systems developed.

The composition of reinforcing fillers significantly affects the properties of the material and the ways it can be used. Glass fibers, as in 3DGF and 3DJGF, have high tensile strength, rigidity, and thermal stability, which allows for use only in those situations where high performance is needed for durability against stress factors such as environment pollution. On the contrary, jute fibers, present in 3DJF, are relatively moderate in strength while being more flexible and cheaper, making them suitable for people-oriented projects that prioritize sustainability as opposed to performance. This helps in enhancing the mechanical properties of jute hybrids without compromising on other aspects, especially environmental issues, allowing performance and sustainability to be achieved in the same application area. This approach paves the way for considering how each different fiber in different percent compositions affects the mechanical and acoustic properties in subsequent studies.

**Figure 1:**
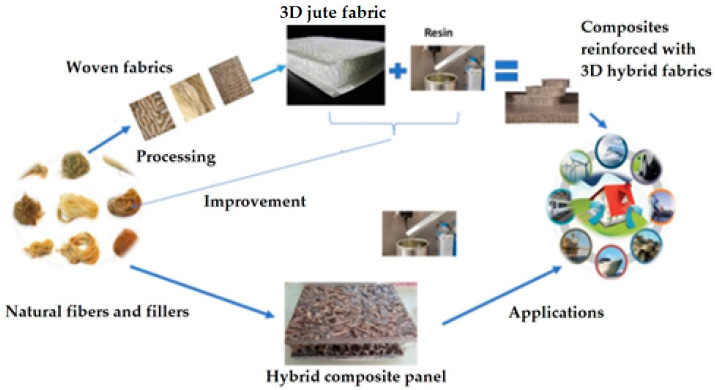
Schematic of the research design.

The overall research design is illustrated in Figure 1.

### 2.2. Methods

#### 2.2.1. 3D Glass Woven Fabric

A 3D glass woven fabric was made of pure E-glass yarns interlaced in three orthogonal directions, producing a completely flexible structure.

#### 2.2.2. 3D Jute Woven Fabric

The jute woven fabric utilizes only pure jute yarns classified as having the same tex of 550 for warp, weft, and pile. The jute yarn increases its fuzziness during the weaving process, which contributes to an increase in adjustments of the grip settings over the settings of glass.

#### 2.2.3. Hybrid Fabric

In this experiment, a hybrid textile was made using E-glass fibers in the warp direction and jute fibers in the weft direction. Jute fibers emerging from the jute bundle may disrupt the distinctive gradient for woven structures by breaking the glass fibers. As the warp tension is increased, the jute fibers in the warp may fray. Thus, the warp sheet tension was maximally reduced, and the back support was adjusted to achieve a strong and straight warp. However, even with alterations made to the fabric construction, inserting glass yarns into the warp direction is still not an easy task due to the fluffiness of the jute yarns.

**Figure 2:**
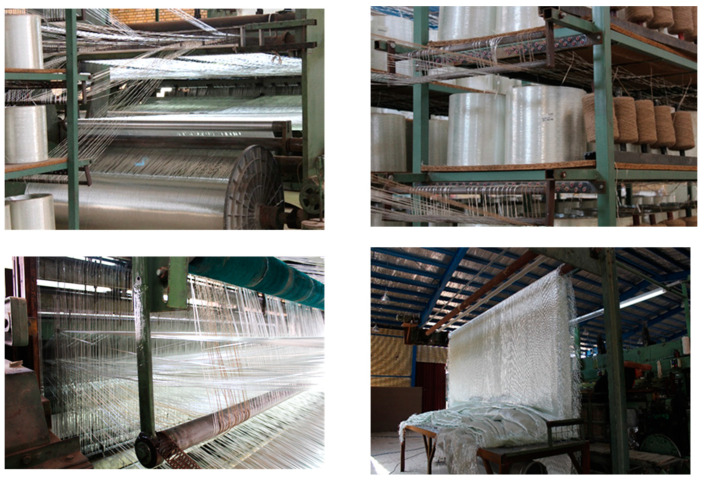
Images of the weaving machine producing 3D woven fabric at Novavaran Sanat Silk Co., Ltd., Kashan, Iran.

Figure 2 shows images of 3D woven fabrics and the weaving machine producing this type of fabric.

**Figure 3:**
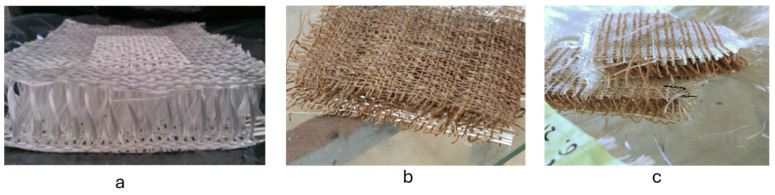
An image of the (**a**) (3DGF), (**b**) (3DJF), and (**c**) (3DJGF).

Removing the phase from the jute fibers during production is vital to prevent unwanted entanglements between warp and weft yarns. The objective of this hybrid design is to optimize mechanical and acoustical performance and be suited for advanced material performance. The samples of 3D woven fabrics from pure E-glass, pure jute, and hybrid glass–jute are shown in Figure 3.

#### 2.2.4. Fabrication of 3D Hybrid Fabrics

The 3D woven hybrid fabric was created by interlacing E-glass yarns along the X direction to form a highly robust structure. The jute yarn was used in the Y and Z directions. In this type of fabric, the delamination failure mode is eliminated in all three directions (X, Y, and Z), and it allows for maximizing the distance of the mass from the center, achieving maximum bending moment tolerance, and significantly increasing the strength-to-weight ratio. For this reason, these fabrics—and the capability to produce them—have become a high-tech advancement limited to a few selected industrial countries (only five countries worldwide).

In these 3D hybrid fabrics, along with the elimination of delamination, the properties of the final composite material can be precisely controlled in all directions by adjusting the material properties of each of the three yarn categories (warp, weft, and pile) and the weaving structure.

The preparation of the composite reinforced with 3D hybrid fabrics involved several critical steps to ensure optimal integration of the fabric layers, resin, and any additional fillers, such as bio-based materials for enhanced properties.

#### 2.2.5. Production Process of 3D Hybrid Composite Panel

**Figure 4:**
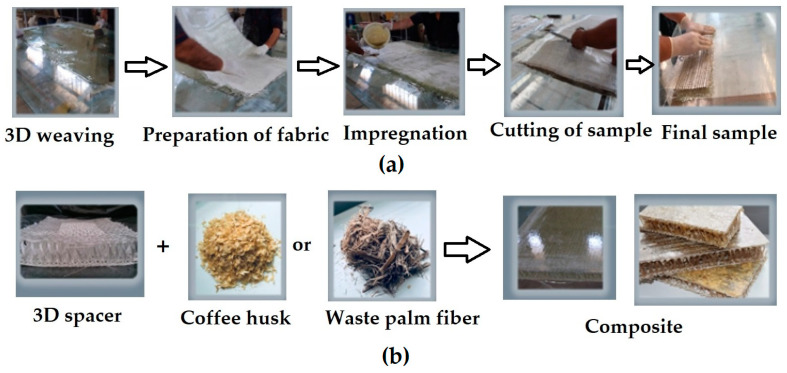
Overall methodology for preparing the 3D spacer composite samples. (**a**) Process flow chart, and (**b**) material flow chart.

To begin the production of 3D hybrid composite panels, the glass workbench was thoroughly cleaned to remove any lint and contaminants. The entire surface of the workbench was then manually coated with a substance called “Poly Wax SV6” (Shimie Afsoon, Golha Blvd., 85 St., Tehranser, Tehran, Iran). This substance was used to facilitate the removal of the composite panel at the end of the process. Figure 4 shows the workbench coated with Poly Wax SV6. Poly Wax SW 6 was applied as the mold release agent due to its excellent non-adherence between composite and mold surface, making demolding easy. It was chosen in accordance with regular processes of the production facility, since it is a substance usually applied for reaching a clean and effective mold release.

Next, a mixture of polyester resin, peroxide acid, and cobalt was prepared. The quantities of each component depend on the dimensions of the desired 3D panel. Now, 50% of the resin mixture (polyester resin, peroxide acid, and cobalt) was applied to the workbench, and the entire surface of the workbench was manually coated with this mixture. Figure 4 shows the workbench surface coated with the resin mixture.

Then, the pre-cut 3D hybrid fabric (cut beforehand using scissors, as it must be placed quickly on the workbench coated with the mixture or else the mixture will dry rapidly) was laid on the resin-coated workbench. After the 3D fabric had been manually impregnated with the mixture, the remaining resin mixture was applied evenly across the entire surface of the 3D hybrid fabric.

The composite panel requires 45 min to fully cure. Once cured, the composite panel was ready for removal from the workbench. The produced panels were kept at ambient temperature for 48 h to complete the curing process. For fabrics containing waste fibers/ bio-fillers, the procedure involves fully absorbing the resin on the surface first, followed by applying waste fibers/fillers on the resin-rich surface. After curing, the samples were cut to the dimensions required for the acoustic test.

The overall methodology is shown in Figure 4.

Samples of three types of fabrics—pure E-glass, pure jute, and hybrid (glass and jute)—were developed. Samples of these three types of fabrics—glass (1), jute (2), and hybrid E-glass and jute (3)—were divided into the following three categories based on the fillers used:

1. With coffee husk fibers.
2. With palm fibers.
3. Without waste fibers/fillers.

**Figure 5:**
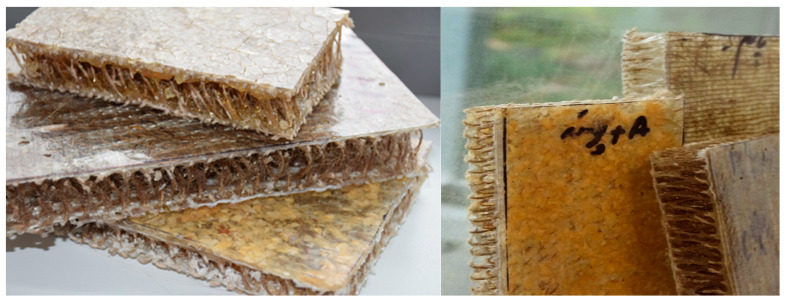
Samples of the produced 3D hybrid composite panels.

Each category was further divided into 6 pieces for performing acoustic tests. The samples of the 3D hybrid composite panels prepared for acoustic testing are shown in Figure 5.

Table 2 shows the geometrical parameters and the ratio of the mixing of fabric samples. It helps in analyzing the influence of the different additives on the final composite structure. The analysis was performed on the finished samples with the horizontal density of the weft equal to 3.2 yarns/cm and the final composite thickness of 2.7 cm. The weight percent ratio of 60:40 (resin to fabric) was maintained in all the samples so that the effect of the additive on the acoustic properties of the composite could be assessed in the absence of varying resin–fabric ratios.

#### 2.2.6. Preparation of Samples for Acoustic Testing

Fifty-four samples of 3D glass fabric/polyester resin composite panels were prepared for testing.

The samples were categorized by reinforcement material into codes: glass yarn (G), jute yarn (J), coffee husk (C), and palm fiber (P). Each sample was sized at 200 × 200 mm. Experiments were conducted in a controlled environment with a constant temperature of 26 °C and a relative humidity of 50%. The sound insulation properties were evaluated using a sound-insulated chamber designed to eliminate external noise interference. The chamber’s thick and foam-lined walls created an anechoic environment ideal for accurate sound transmission measurements.

**Figure 6:**
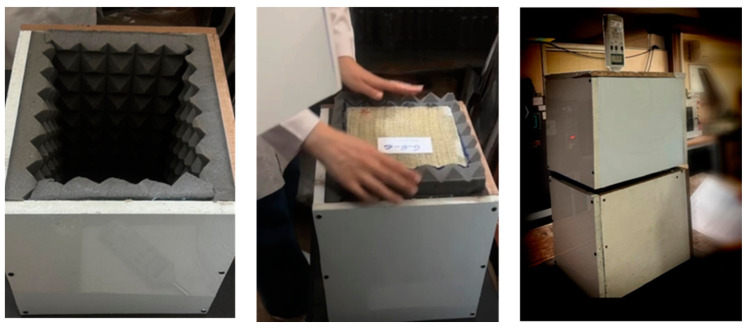
View of the sound measuring device (a chamber to act as an obstacle to sound transmission) in the laboratory.

Each sample was centrally placed in the chamber, serving as a barrier between the sound source and the detector. The distance between the sound source and the lower surface of the sample was fixed at 224 mm. The sound intensity was initially recorded without any sample to establish a reference level. The sample was introduced as a barrier, and the sound intensity was measured on the opposite side. Measurements were taken at two frequencies, 1000 Hz and 10,000 Hz. Each test was repeated three times, with the average value reported to ensure precision. To account for the samples’ non-homogeneous thickness and honeycomb design, foam insulators were applied to the cut edges to prevent sound leakage during testing. The sound absorption measurement is demonstrated in Figure 6.

#### 2.2.7. Principle of Sound Insulation Measurement

Sound insulation was assessed by measuring the difference in sound pressure levels (SPL) on both sides of the sample when subjected to sound waves. The sound transmission loss (STL) or sound reduction index (SRI) was calculated as follows:

STL = (L1) − (L2) (1)

where:

L1 = sound level on the source side (dB).
L2 = sound level on the receiving side (dB).

Higher STL values indicate better sound insulation. Sound frequency plays a crucial role in the material’s insulation properties. In this study, sound insulation performance was evaluated at two frequencies, 1000 Hz and 10,000 Hz.

## 3. Results and Discussion

The test results are shown in Table 3, Table 4 and Table 5 below. The intensity of the sound in the laboratory environment was 40.2 dB.

**Figure 7:**
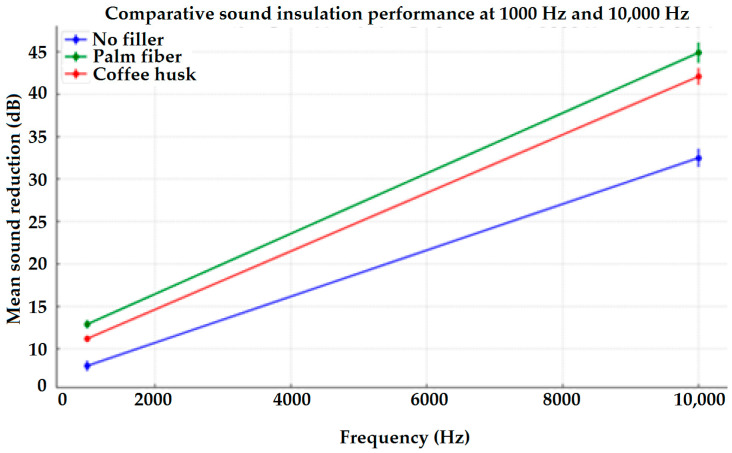
Comparative line graph illustrating the sound insulation performance of different materials (no filler, palm fiber, and coffee husk) at two frequencies, 1000 Hz and 10,000 Hz. The error bars around each point represent data variability and uncertainty at each frequency.

The sound insulation test results shown in the tables provide detailed data on the frequency (Hz), the sound intensity measured in the presence of the sample (dB), the sound intensity measured without the sample (dB), and the resulting sound reduction level (dB). More importantly, the tables help in demonstrating the performance of different fabric samples in the reduction of noise transmission at varying frequencies. The sound intensity is measured with the use of a sample and also without the sample. Hence, the value of the sound reduction level shows how well each type of composite can reduce sound at different frequency levels. Figure 7 shows a comparative account of the sound insulation performance of different materials (no filler, palm fiber, and coffee husk) at two different frequencies, 1000 Hz and 10,000 Hz. The error bars around each point represent data variability and uncertainty at each frequency.

### Data Analysis

In this investigation, the sound insulation was determined by calculating the difference between sound levels without the samples versus sound levels with the samples. The difference was averaged by trials to determine the performance of sound insulating properties of each material type. One of the protective chamber devices was used to measure sound intensity, and further analysis was conducted with Statistical Package for Social Sciences (SPSS) software, v2024. In this phase of the study, 54 different samples of 3D woven glass, 3D woven jute, and 3D hybrid composite panels of glass/polyester resin with different measured characteristics were characterized. Therefore, the effect of the composition on sound reduction levels was evaluated statistically. Three repetitions for the test of each sample were performed as guided by prior literature [35,37].

Considering the standard deviation of the data obtained from acoustic tests and dividing them by the means of the data, the coefficient of variation (CV%) was calculated (the spread of data around the mean). Calculations showed that in almost all cases, the coefficient of variation of the data was less than 7%, and, in most cases, it was lower than 5%. Therefore, in the statistical analysis of data, this number of samples can be considered a normal population. Therefore, to compare means and detect significant differences or effects of influential parameters, independent sample T-test and one-way ANOVA were used. These statistical analyses were performed in SPSS version 22 (IBM, Chicago, IL, USA). As shown in the experimental investigation, in addition to the type of yarn used in three-dimensional fabric composites and the use of waste fibers/fillers from cellulose, the effect of many other parameters on the acoustic properties of the resulting composites has been measured. These parameters include (a) density, (b) thickness, and (c) frequency of emitted sound. Thus, in these statistical tests, to measure the effect of the type of yarn used in three-dimensional fabric composites and the use of palm fiber and coffee husk on the surface of these composites, each test was carried out under the stable conditions of each of the mentioned triple parameters. This was also performed by filtering the data using the select cases command in SPSS. In this way, it can be claimed that in the statistical analysis, the interference and influence of other parameters apart from the two parameters of interest in the dependent variable, which account for the acoustic property of the substance, have been prevented. All validations were performed at a confidence level of 95% and based on the sigma value in the output of the software analysis.

Table 6 and Table 7 show the output of the statistical analysis for the effect of type of 3D woven fabric composites, type of filler (palm fiber, coffee husk, or none), and frequencies of 1000 and 10,000 Hz.

Table 8, Table 9, Table 10, Table 11 and Table 12 show the output of the software for the effect of waste fiber/filler type, such as palm fiber and coffee husk, on the sound absorption of the 3D woven fabric composite type for these two frequencies.

From the results obtained through one-way ANOVA, partial eta squared (η^2^) was computed as the measure of effect size that provided insight into strength across different composite configurations. The analysis showed that composites with palm fiber have a much higher effect size, η^2^, when compared with the composites prepared either with coffee husk or without fillers, for sound reduction at both frequencies 1000 Hz and 10,000 Hz. In its specific case, the average values for the composite with palm fiber at 10,000 Hz frequency were higher, presenting about 43.6 dB of sound reduction and yielding a robust effect size, evidencing its highest capability for noise attenuation.

Only by presenting such effect sizes in the results a better understanding of the effectiveness of each type of composite panel in real-world acoustic applications will be appreciated, thus further reinforcing the relevance of bio-based fillers like palm fiber.

Overall, considering the diversity of the triple gender yarns woven into the structure of three-dimensional fabric and examining the acoustic properties of composite panels in three surface covering conditions and dual frequencies of 1000 and 10,000 Hz, a total of 54 statistical tests needed to be conducted (assuming constant diameter and density in all samples).

All statistical tests indicated a significant influence of using natural fiber yarns in the structure of hybrid composite panels on their acoustic properties, especially in cases where these yarns were used in the upper and lower layers as well as in the pile (JJJ state).

In other words, the effect of natural fiber yarns (jute) as part of the pile alongside glass yarns, both individually and in all X, Y, and Z directions, will significantly enhance the overall acoustic properties of the composite panels. This impact is very tangible at both mentioned frequencies and with any type of surface coating.

The statistical tests conducted on the effect of surface coatings/fillers of composite with waste fibers, palm fiber and coffee husk, and without any surface coating also demonstrated the positive and significant impact of these coatings/fillers on enhancing the acoustic properties of these composites. In some cases, the greatest impact was related to the use of coffee husk waste, while in others, despite the significant impact of surface coating used, no significant effect was found for these two types of fillers on the acoustic properties of the composite panels.

**Figure 8:**
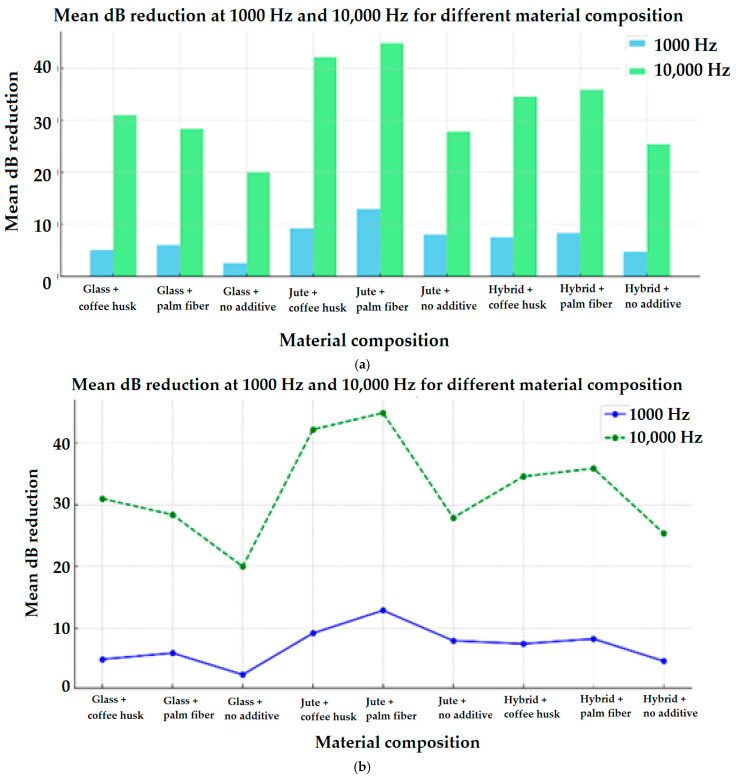

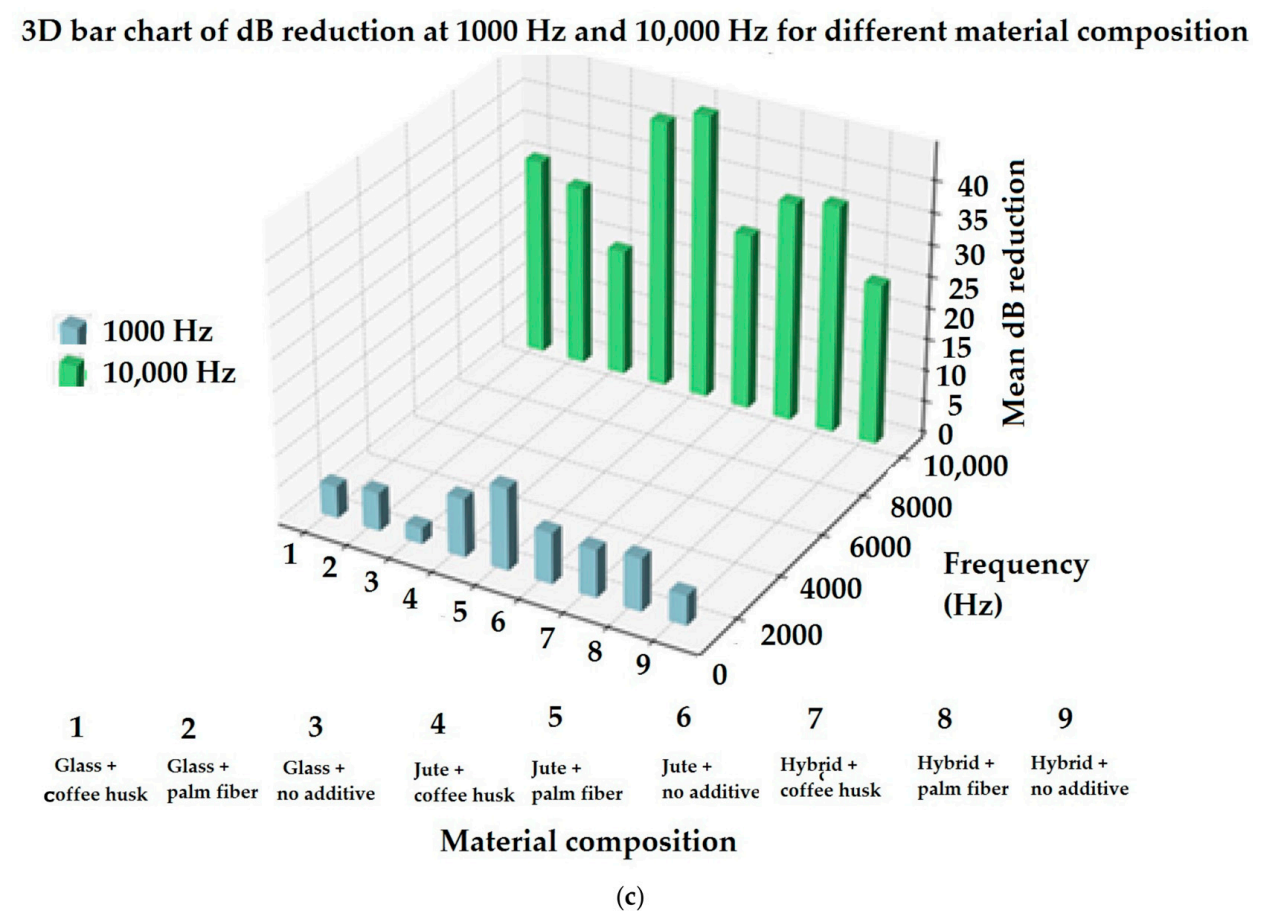
(**a**) Bar chart, (**b**) line chart, and (**c**) 3D bar chart illustrating the sound reduction levels for different materials at both 1000 Hz and 10,000 Hz frequencies.

The graphs shown in Figure 8 indicate the averages of these significant effects for the different types of yarns used in the three directions (X, Y, and Z), as well as for the use or non-use of the mentioned surface coatings/fillers at 1000 Hz as well as 10,000 Hz frequencies.

As shown in Figure 8, the highest sound absorption was observed in the 3D woven jute composites with additive of waste palm fiber, which outperformed the other samples. This enhanced performance can be attributed to the superior sound-absorbing qualities of natural fibers such as jute, combined with the scattering effect of waste palm fibers, which is responsible for the porous surface of the material. Jute fibers exhibit better damping of vibrations compared to synthetic materials, which is a key factor in their higher sound absorption capacity.

When comparing the effect of coffee husk and palm fiber as biomass fillers, both exhibited notable improvements in sound insulation, but the palm fiber generally performed better across different samples. This could be due to its inherent structural characteristics, which contribute to a more effective sound barrier. Palm fiber, being more porous and less dense than coffee husk, may facilitate a higher degree of sound wave entrapment, leading to better insulation. Although panels containing palm fiber additives appeared to reduce sound more than those containing coffee husk, statistical analysis revealed no discernible difference between the two, indicating that both are efficient and eco-friendly fillers for soundproofing applications.

## 4. Conclusions

As a result, this investigation showed that the 3D hybrid woven fabric composites enhanced the sound insulation properties substantively involving natural fibers such as jute and biomass, e.g., coffee husk and waste palm fibers. Amongst these materials, 3DJF exhibited substantial sound absorption at a higher frequency with a maximum value of 44.9 dB at 10,000 Hz. The 3DJGF material also showed good performance, in particular with waste palm fibers, in which the sound was absorbed by 35.9 dB at 10,000 Hz. Comparatively, the 3DGF composites provided a lower level of acoustic performance but still performed satisfactorily. The highest sound absorption was observed in the 3D woven jute composites with additive of waste palm fiber, which outperformed the other samples. Palm fiber, being more porous and less dense than coffee husk, may facilitate a higher degree of sound wave entrapment, leading to better insulation.

From the one-way ANOVA, it was proved that fiber composition in the 3D spacer fabric and additive fillers have a considerable influence on soundproofing, with *p*-values less than 0.05.

The use of jute and biomass fillers like palm fiber and coffee husk in place of synthetic materials has compositional benefits. This method minimizes waste since agro-based by-products are used, but also reduces the reliance on synthetic materials, leading to a reduced environmental impact from the manufacture of synthetic material. Moreover, the composites prepared from the above materials possess potential biodegradability and, thus, support their sustainability value to be used in suitable and sustainable soundproofing applications. These features enable sound-absorbing materials to play a role in alleviating environmental challenges and promoting sustainability for the industry. This information strengthened the overall perspective that eco-friendly materials derived from natural resources and waste fibers could be more beneficial in the sound insulation field, providing sustainable solutions to industries ranging from interior design of house buildings to automotive and constructions at airports.

## Figures and Tables

**Table 1:** Structural properties and type of the yarns and fabrics.

Fabric Type	Thickness (mm)	Weft Density (/cm)	E-Glass Yarn Count (Tex)	Jute Yarn Count (Tex)	Pile Yarn	Warp	Weft
3DGF	2.7	3.2	600	-	-	Glass	Glass
3DJF	2.7	3.2	-	551.18	Jute	Jute	Jute
3DJGF	2.7	3.2	600	551.18	jute	Glass	Glass

**Table 2:** Details of geometrical parameters of samples and mixing ratio.

Sample Code	Fabric Type Code	Additive	Weft Density	Final Composite Thickness	Weight Ratio (Resin–Fabric)
1	3D glass (3DGF)	Palm fiber	3.2	2.7 cm	60:40
1	3D glass (3DGF)	Coffee husk	3.2	2.7 cm	60:40
1	3D glass (3DGF)	Without additives	3.2	2.7 cm	60:40
2	3D jute (3DJ)	Palm fiber	3.2	2.7 cm	60:40
2	3D jute (3DJ)	Coffee husk	3.2	2.7 cm	60:40
2	3D jute (3DJ)	Without additives	3.2	2.7 cm	60:40
3	Hybrid (3DJGF)	Palm fiber	3.2	2.7 cm	60:40
3	Hybrid (3DJGF)	Coffee Husk	3.2	2.7 cm	60:40
3	Hybrid (3DJGF)	Without additives	3.2	2.7 cm	60:40

**Table 3:** Results of sound insulation test for glass fabric composite panels.

Sample No.	Sample Code	Sample Type	Frequency (Hz)	Sound Intensity Measured in the Presence of Sample (dB)	Sound Intensity Measured Without Sample (dB)	Sound Reduction Level (dB)
1	G-G-G (C1)	G-G-G + coffee husk	1000	76.1	81.1	5.0
2	G-G-G (C2)		1000	75.9	81.1	5.2
3	G-G-G (C3)		1000	76.0	81.1	5.1
4	G-G-G (C1)		10,000	57.1	88.1	31.0
5	G-G-G (C2)		10,000	57.1	88.1	31.0
6	G-G-G (C3)		10,000	57.5	88.1	30.6
7	G-G-G (P1)	G-G-G + palm fiber	1000	75.1	81.1	6.0
8	G-G-G (P2)		1000	75.7	81.1	5.4
9	G-G-G (P3)		1000	75.6	81.1	5.5
10	G-G-G (P1)		10,000	59.7	88.1	28.4
11	G-G-G (P2)		10,000	59.2	88.1	28.9
12	G-G-G (P3)		10,000	54.4	88.1	33.7
13	G-G-G (1)	G-G-G (no filler)	1000	78.6	81.1	2.5
14	G-G-G (2)		1000	78.5	81.1	2.6
15	G-G-G (3)		1000	78.6	81.1	2.5
16	G-G-G (1)		10,000	68.1	88.1	20.0
17	G-G-G (2)		10,000	68.0	88.1	20.1
18	G-G-G (3)		10,000	68.2	88.1	19.9

**Table 4:** Results of sound insulation test for glass–jute hybrid fabric composite panels.

Sample No.	Sample Code	Sample Type	Frequency (Hz)	Sound Intensity Measured in the Presence of Sample (dB)	Sound Intensity Measured Without Sample (dB)	Sound Reduction Level (dB)
19	G-J-G (C1)	G-J-G + coffee husk	1000	73.6	81.1	7.5
20	G-J-G (C2)		1000	73.2	81.1	7.9
21	G-J-G (C3)		1000	74.1	81.1	7.0
22	G-J-G (C1)		10,000	53.5	88.1	34.6
23	G-J-G (C2)		10,000	54.2	88.1	33.9
24	G-J-G (C3)		10,000	53.5	88.1	34.6
25	G-J-G (P1)	G-J-G + palm fiber	1000	73.8	81.1	7.3
26	G-J-G (P2)		1000	72.8	81.1	8.3
27	G-J-G (P3)		1000	72.7	81.1	8.4
28	G-J-G (P1)		10,000	53.7	88.1	34.4
29	G-J-G (P2)		10,000	52.2	88.1	35.9
30	G-J-G (P3)		10,000	52.6	88.1	35.5
31	G-J-G (1)	G-J-G (no filler)	1000	76.4	81.1	4.7
32	G-J-G (2)		1000	76.3	81.1	4.8
33	G-J-G (3)		1000	76.4	81.1	4.7
34	G-J-G (1)		10,000	73.6	88.1	25.4
35	G-J-G (2)		10,000	73.2	88.1	25.6
36	G-J-G (3)		10,000	68.2	88.1	24.1

**Table 5:** Results of sound insulation test for jute fabric composite panels.

Sample No.	Sample Code	Sample Type	Frequency (Hz)	Sound Intensity Measured in the Presence of Sample (dB)	Sound Intensity Measured Without Sample (dB)	Sound Reduction Level (dB)
37	J-J-J (C1)	J-J-J + coffee husk	1000	71.9	81.1	9.2
38	J-J-J (C2)		1000	70.2	81.1	10.9
39	J-J-J (C3)		1000	70.0	81.1	11.1
40	J-J-J (C1)		10,000	45.9	88.1	42.2
41	J-J-J (C2)		10,000	46.6	88.1	41.5
42	J-J-J (C3)		10,000	46.3	88.1	41.8
43	J-J-J (P1)	J-J-J + palm fiber	1000	68.2	81.1	12.9
44	J-J-J (P2)		1000	70.3	81.1	10.8
45	J-J-J (P3)		1000	70.1	81.1	11.0
46	J-J-J (P1)		10,000	43.2	88.1	44.9
47	J-J-J (P2)		10,000	43.3	88.1	44.8
48	J-J-J (P3)		10,000	46.9	88.1	41.2
49	J-J-J (1)	J-J-J (no filler)	1000	73.1	81.1	8.0
50	J-J-J (2)		1000	73.5	81.1	7.6
51	J-J-J (3)		1000	73.8	81.1	7.3
52	J-J-J (1)		10,000	60.2	88.1	27.9
53	J-J-J (2)		10,000	58.4	88.1	29.7
54	J-J-J (3)		10,000	58.5	88.1	29.6

**Table 6:** Result from one-way ANOVA: effect of material composition (MC) on dB difference for 1000 Hz frequency.

Material Composition (MC)	Additive	Mean dB Difference	Standard Deviation	Significance (*p*-Value)
Glass fiber (G-G-G)	Palm fiber	6.0	0.4	*p* < 0.05
Glass fiber (G-G-G)	Coffee husk	5.1	0.3	*p* < 0.05
Glass fiber (G-G-G)	None	2.5	0.2	*p* < 0.05
Jute fiber (J-J-J)	Palm fiber	12.9	0.5	*p* < 0.01
Jute fiber (J-J-J)	Coffee husk	11.1	0.4	*p* < 0.01
Jute fiber (J-J-J)	None	8.0	0.6	*p* < 0.05
Hybrid (G-J-G)	Palm fiber	7.5	0.3	*p* < 0.01
Hybrid (G-J-G)	Coffee husk	7.2	0.4	*p* < 0.01
Hybrid (G-J-G)	None	4.7	0.5	*p* < 0.05

**Table 7:** Result from one-way ANOVA: effect of material composition (MC) on dB difference for 10,000 Hz frequency.

Material Composition (MC)	Additive	Mean dB Difference	Standard Deviation	Significance (*p*-Value)
Glass fiber (G-G-G)	Palm fiber	28.4	0.7	*p* < 0.05
Glass fiber (G-G-G)	Coffee husk	31.0	0.5	*p* < 0.05
Glass fiber (G-G-G)	None	19.9	0.8	*p* < 0.05
Jute fiber (J-J-J)	Palm fiber	44.9	1.2	*p* < 0.01
Jute fiber (J-J-J)	Coffee husk	41.8	0.9	*p* < 0.01
Jute fiber (J-J-J)	None	29.6	1.1	*p* < 0.05
Hybrid (G-J-G)	Palm fiber	35.9	0.9	*p* < 0.05
Hybrid (G-J-G)	Coffee husk	34.6	1.0	*p* < 0.05
Hybrid (G-J-G)	None	25.4	1.2	*p* < 0.05

**Table 8:** Result from one-way ANOVA: effect of waste fiber/filler (palm fiber, coffee husk) on 3D hybrid glass–jute composite (3DGJC) at 1000, 10,000 Hz.

Material Composition (MC)	Additive	Mean dB Difference	Standard Deviation	Significance (*p*-Value)
Glass fiber (G-G-G)	Palm fiber	28.4	0.7	*p* < 0.05
Glass fiber (G-G-G)	Coffee husk	31.0	0.5	*p* < 0.05
Glass fiber (G-G-G)	None	19.9	0.8	*p* < 0.05
Jute fiber (J-J-J)	Palm fiber	44.9	1.2	*p* < 0.01
Jute fiber (J-J-J)	Coffee husk	41.8	0.9	*p* < 0.01
Jute fiber (J-J-J)	None	29.6	1.1	*p* < 0.05
Hybrid (G-J-G)	Palm fiber	35.9	0.9	*p* < 0.05
Hybrid (G-J-G)	Coffee husk	34.6	1.0	*p* < 0.05
Hybrid (G-J-G)	None	25.4	1.2	*p* < 0.05

**Table 9:** Result from one-way ANOVA: effect of waste fiber/filler (palm fiber, coffee husk, none) on 3D Hybrid glass–jute composite (3DGJC) at 1000 Hz.

Waste Fiber Type	Mean dB Difference	Standard Deviation	Significance (*p*-Value)
Palm fiber	12.9	0.7	*p* < 0.01
Coffee husk	11.1	0.5	*p* < 0.01
None	8.0	0.6	*p* < 0.05

**Table 10:** Result from one-way ANOVA: effect of waste fiber/filler (palm fiber, coffee husk, none) on 3D Hybrid glass–jute composite (3DJGC) at 1000 Hz and 10,000 Hz.

Frequency (Hz)	Waste Fiber Type	Mean dB Difference	F-Value	*p*-Value
1000 Hz	Palm fiber	7.5	15.8	0.01
1000 Hz	Coffee husk	7.2	14.6	0.01
1000 Hz	None	4.7	10.5	0.02
10,000 Hz	Palm fiber	35.9	45.7	0.001
10,000 Hz	Coffee husk	34.6	42.8	0.001
10,000 Hz	None	25.4	32.5	0.01

**Table 11:** Result from one-way ANOVA: effect of waste fiber/filler on 3D jute fabric composite (3DJFC).

Frequency (Hz)	Waste Fiber Type	Mean dB Difference	F-Value	*p*-Value
1000 Hz	Palm fiber	12.9	17.2	0.005
1000 Hz	Coffee husk	11.1	15.6	0.008
1000 Hz	None	8.0	12.1	0.03
10,000 Hz	Palm fiber	44.9	50.5	0.0005
10,000 Hz	Coffee husk	41.8	47.8	0.0006
10,000 Hz	None	29.6	39.5	0.001

**Table 12:** Result from one-way ANOVA: effect of waste fiber/filler on 3D glass fabric composite (3DGFC).

Frequency (Hz)	Waste Fiber Type	Mean dB Difference	F-Value	*p*-Value
1000 Hz	Palm fiber	6.0	11.5	0.02
1000 Hz	Coffee husk	5.1	10.8	0.03
1000 Hz	None	2.5	8.5	0.04
10,000 Hz	Palm fiber	28.4	35.2	0.002
10,000 Hz	Coffee husk	31.0	37.1	0.001
10,000 Hz	None	19.9	30.5	0.005

## Data Availability

The original contributions presented in this study are included in the article. Further inquiries can be directed to the corresponding authors.
